# Challenges in Access and Utilization of Sexual and Reproductive Health Services Among Youth During the COVID-19 Pandemic Lockdown in Uganda: An Online Cross-Sectional Survey

**DOI:** 10.3389/frph.2021.705609

**Published:** 2022-02-03

**Authors:** Simon Binezero Mambo, Franck Katembo Sikakulya, Robinson Ssebuufu, Yusuf Mulumba, Henry Wasswa, Solomon Adomi Mbina, Jean Christophe Rusatira, Fiona Bhondoekhan, Louis K. Kamyuka, Surat Olabisi Akib, Claude Kirimuhuzya, Jane Nakawesi, Patrick Kyamanywa

**Affiliations:** ^1^Department of Public Health, School of Allied Health Sciences, Kampala International University Western Campus, Kampala, Uganda; ^2^Youth Alliance for Reproductive Health, Goma, Democratic Republic of the Congo; ^3^Department of Surgery, Faculty of Clinical Medicine and Dentistry, Kampala International University Western Campus, Kampala, Uganda; ^4^Faculty of Medicine, Université Catholique du Graben, Butembo, Democratic Republic of the Congo; ^5^Biostatistics, Cancer Institute, Makerere University, Kampala, Uganda; ^6^Reproductive Health Uganda, Iganga, Uganda; ^7^Johns Hopkins Bloomberg School of Public Health, Bill & Melinda Gates Institute for Population and Reproductive Health, Baltimore, MD, United States; ^8^Department of Epidemiology, Johns Hopkins Bloomberg School of Public Health, Baltimore, MD, United States; ^9^Department of HIV/TB (CHAI Clinic), Kampala International University Teaching Hospital, Kampala, Uganda; ^10^Department of Pathology, Faculty of Clinical Medicine and Dentistry, Kampala International University Teaching Hospital, Kampala, Uganda; ^11^Department of Pharmacology and Toxicology, School of Pharmacy, Kampala International University Western Campus, Kampala, Uganda; ^12^Department of Paediatrics, Mildmay Uganda Hospital, Wakiso, Uganda

**Keywords:** sexual reproductive health, COVID-19, youth, Uganda, lockdown

## Abstract

**Introduction:**

Sexual and Reproductive Health access to Information services is still a pressing need for youth in Uganda even during the COVID-19 pandemic, which has disrupted health care access in many countries. The aim of this study was to explore the challenges in access and utilization of sexual and reproductive health services as faced by youth during the COVID-19 pandemic lockdown in Uganda.

**Methods:**

This was a cross-sectional study carried out from 28th April 2020 to 11th May 2020 in Uganda. An online questionnaire was disseminated to youth aged between 18 and 30 years over a period of 14 days. The snowball sampling method was used to recruit participants. STATA version 14.2 was used for statistical analysis.

**Results:**

Of 724 participants, 203 (28%) reported that they did not have access to information and/or education concerning sexual and reproductive health (SRH). More than a quarter of the participants (26.9%, *n* = 195) reported that testing and treatment services of sexually transmitted infections were not available during the lockdown, and 27.2% could not obtain contraceptive supplies. Access to HIV/AIDS care services and menstrual supplies was also impaired. Lack of transportation was the commonest factor cited as limiting access to SRH services during the lockdown (68.7%), followed by the long distance from home to SRH facilities (55.2%), high cost of services (42.2%) and the curfew (39.1%). Sexually transmitted infections were the commonest SRH problems related to SRH during the lockdown (40.4%) followed by unwanted pregnancy (32.4%) and sexual abuse (32.4%). Marital, educational, and employment status were significantly correlated with the reported experiences of the participants.

**Conclusion:**

Access to SRH information and services for Ugandan youth was restricted during the COVID-19 lockdown and leaving them vulnerable to various SRH risks and adverse outcomes. Lack of transportation, long distances to health facilities, and high cost of services were important limiting factors. The Government and other stakeholders should incorporate SRH among the priority services to be preserved during future outbreaks.

## Introduction

On 11th March, 2020, the World Health Organization (WHO) declared COVID-19 to be a pandemic (1). As of 17 October 2021, 99,363,697 cases of COVID-19 had been confirmed worldwide with 2,135,959 deaths. That report included 2,489,430 cases and 24,464 deaths in Africa. By that date, Uganda had registered 39,188 confirmed cases of COVID-19 with 318 reported deaths (2). Many governments responded to the pandemic by instituting mass quarantine, lockdown, and/or social distancing (3). Uganda announced a lockdown and dawn-to-dusk curfew on 20th March 2020. Subsequently other COVID-19 pandemic control measures were introduced, all of which have severely curtailed access to sexual and reproductive health services with direct and indirect consequences for young people (4).

As quarantines and school closures were put in place to contain the spread of disease in many countries across the world (1, 4, 5), women and adolescent girls became more vulnerable to SRH problems such as coercion, exploitation, sexual abuse, and restricted access to contraception. Delays in the care of pregnant women and an increase in the number of unsafe abortions have also been reported (6, 7). The United Nations Population Fund (UNFPA) in its COVID-19 Pandemic Global Response Plan emphasized that SRH is a significant public health issue that demands urgent and sustained attention and investment (8). Similarly, the Inter-Agency Working Group (IAWG) on reproductive health has also recommended that comprehensive sexual and reproductive health services should be maintained (6).

The Government of Uganda issued directives to protect pregnant women's access to maternity services by allowing for transportation of pregnant women seeking care and access to call-in ambulance services provided by the Ministry of Health (7). However, other sexual and reproductive health services were not a priority during the lockdown, resulting in diminished access to contraceptive and menstrual health supplies, and a reduction in SRH programs for comprehensive sex education, reduction of gender-based violence and support for victims of assault (7). The reallocation of already limited resources to deal with the pandemic and the re-assignment of health care workers from their usual duties limits the capacity to provide other essential SRH services, leading to adverse outcomes of chronic health conditions, disabilities, HIV, and pregnancy (9, 10). Reports are emerging of a rise in gender-based violence, unwanted pregnancy among young girls, unsafe abortion, closure of antenatal care services in some of the public health facilities, and a sharp decline in women and girls seeking SRH services (7). Youth-friendly corners at health facilities that had been established by the Ministry of Health of Uganda and donors to make SRH services more accessible were not available during the lockdown (7).

Uganda has a national adolescent health policy that aims to streamline adolescent health concerns into the national development process to improve youths' quality of life and standard of living (11). Even before the COVID-19 pandemic, SRH services for young people in Uganda were inadequate (12, 13). Studies have identified young people in Uganda and other sub-Saharan African countries as having limited access to contraception, and a lack of staff trained to address the sexual health needs and education gaps of youth. These deficits have negatively impacted upon youth's sexual and reproductive health include sexually transmitted infections (STIs), defilement, rape, substance abuse, and unwanted pregnancies which majority end into unsafe abortions (12, 14). The Uganda demographic health survey of 2016 highlights a 28% unmet family planning need and a prevalence of over 25% teenage pregnancies among sexually active young people by the age of 16 years (15, 16). Unintended pregnancy is common in Uganda with the attendant high levels of unplanned births, unsafe abortions, and maternal injury and death (15, 16).

This study was carried out to explore the challenges faced by youth in accessing and utilizing sexual and reproductive health services during the first wave of COVID-19 pandemic lockdown in Uganda, and to inform appropriate intervention measures to respond to young people's sexual and reproductive health needs during pandemics and other health emergencies.

## Methods

### Study Population

The Uganda youth policy defines youth as all young persons, aged 12–30 years (17). This age group includes more than 10 million people, 22.9% of the Ugandan population (18). This study was focused on Ugandan youth of consent age of 18 years and above at the time of the study.

In Uganda, ~78% of its population is below 30 years of age (19) and despite relatively high costs of voice and data services, nearly 71% of Ugandans own mobile phones, and the coverage of internet-enabled handsets is increasing (20).

### Study Design and Setting

A nationwide cross-sectional online survey was conducted among Ugandan youth from 28th April to 11th May 2020.

### Data Collection and Instrument

An online questionnaire was developed from a validated and published study on SRH needs and rights of young people in Uganda (21) and was composed of 22 questions. Six questions were related to socio-demographics (age, sex, marital status, educational level, location, occupation). Twelve questions addressed access to SRH information and services during the COVID-19 lockdown. Two questions dealt with factors that limited access to SRH information and services and two questions asked about SRH problems during the COVID-19 lockdown (Supplementary Table 1). As the study was conducted online, participation in the study required internet access, minimal computer literacy level, and ability to operate WhatsApp, Twitter, or Facebook. To ensure correctness and appropriateness to the local context, the questionnaire was pretested and reviewed by two independent reviewers and piloted in Bushenyi district of western Uganda whose responses were not included in the study.

To calculate the sample size, we hypothesized that at 99% confidence interval at 5% margin of error for a population of 10,326,072 Ugandan youth aged 18–30 years (21), 50% of the respondents would have a challenge related to SRH during the first wave of the COVID-19 pandemic lockdown. Using the Open-Source Epidemiologic Statistics for Public Health (OpenEpi), v.3.01 (Dean AG, Sullivan KM, Soe MM. Open-Epi: www.OpenEpi.com, updated April 06, 2013), a minimum sample size of 664 respondents was needed in this study. As the country was under lockdown, social media (WhatsApp, Twitter, or Facebook) was used to conduct the survey. A snowball sampling technique was used to pool the initial eligible respondents who were in turn encouraged to recruit more respondents from their acquaintances in different regions of the country by forwarding to them the link to the survey. The questionnaire was administered for a period of 14 days. On receiving and clicking on the link, participants were auto-directed to an informed consent. Completion of this page was required in order to proceed to the questionnaire. At the end of the data collection period, 724 participants had responded to the study tool from the four regions of the country (274 in Central region, 254 in Western region, 122 in Eastern region, and 74 in Northern).

### Data Processing and Analysis Plan

Categorical variables were presented using frequencies, and/or figures whereas continuous variables were presented using means, standard deviations (SD). Bivariate and multivariate regression analyses were used to investigate association of socio-demographics with factors that influenced access to SRH as well as the experienced problems in SRH during the COVID-19 lockdown. Results are presented as Crude Prevalence Ratios (CPR) and Adjusted Prevalence Ratios (APR), respectively. We used the Poisson Regression with Robust standard error option, which is appropriate for cross-sectional studies (22). STATA version 14.2 (StataCorp, College Station, Texas, USA) was used for statistical analysis.

## Results

A total of seven hundred thirty-three (733) participants completed the online questionnaire. Nine (9) participants were excluded from the survey because they were above 30 years of age. Responses from seven hundred twenty-four (724) participants were analyzed.

### Socio-Demographic Characteristics of Participants

Males represented 56.4% of participants. The mean age was of 24.4 (SD ±2.8) years. The majority (78.0%) of participants were single and 87.2% had attained an educational level of university. Only 27.2% were salaried employees. Most participants were from central Uganda (37.8%) followed by western Ugandan (35.1%) (Table 1).

**Table 1:** Socio-demographic characteristics of study participants.

**Variable**	**Frequency *N* (%)**
Sample size	724 (100)
Sex	
Female	316 (43.6)
Male	408 (56.4)
Age group in years	
18–24	395 (54.6)
25–30	329 (45.4)
Marital status	
Living single	555 (78.0)
Married	81 (11.2)
Cohabiting	78 (10.8)
Education level	
University	631 (87.1)
Vocational or technical institution	46 (6.4)
Secondary school and below	47 (6.5)
Location/region in Uganda	
Central Uganda	274 (37.8)
Western Uganda	254 (35.1)
Eastern Uganda	122 (16.9)
Northern Uganda	74 (10.2)
Employment status	
Students	337 (46.5)
Paid employment (employee on a salary)	197 (27.2)
Self-employed (business/income generating activity)	62 (8.6)
Unemployed: no structured activity	69 (9.5)
Unemployed: volunteer or unpaid work	59 (8.1)

### Access to Sexual and Reproductive Health Services of Participants During the COVID-19 Lockdown

Table 2 shows the reported availability of SRH services to the participants in this study. Out of 724 participants, 203 (28.0%) reported no access to information on SRH. Regarding STI's, 195 participants (26.9%) reported that they did not have access to testing and treatment for STIs while 29.6% participants did not know whether such services and information were available. About a third (27.2%) of participants did not have easy access to their preferred modern method of contraception during the COVID-19 lockdown. Inability to access HIV testing and counseling services was reported by 22% of participants.

**Table 2:** Access to sexual and reproductive health services among Ugandan youth during the COVID-19 lockdown.

**Variables**	**All (%)** ***n* = 724**
**Accessibility to information and/or education concerning sexuality**	
No	203 (28.0%)
Yes	521 (72.0%)
**Accessibility to testing and treatment services of STIs**	
No	195 (26.9%)
Yes	315 (43.5%)
Don't know	214 (29.6%)
**Accessibility to the preferred modern contraceptive**	
Not easily	197 (27.2%)
Easily	132 (18.2%)
Not applicable	395 (54.6%)
**Availability of HIV testing and counseling services**	
No	159 (22%)
Yes	349 (48.2%)
I don't know	216 (29.8%)
**Access to antiretroviral therapy (medication)**	
Not easily	50 (6.9%)
Easily	12 (1.7%)
Not applicable	662 (91.4%)
**Access to menstrual health products such as sanitary pads**	
Not easily	127 (17.5%)
Easily	189 (26.1%)
Not applicable	408 (56.4%)
**Availability of pregnancy care**	
Yes	36 (81.8%)
No	8 (18.2%)
**Access to post abortion care services**	
Yes	19 (79.2%)
No	5 (20.8%)

**Figure 1:**
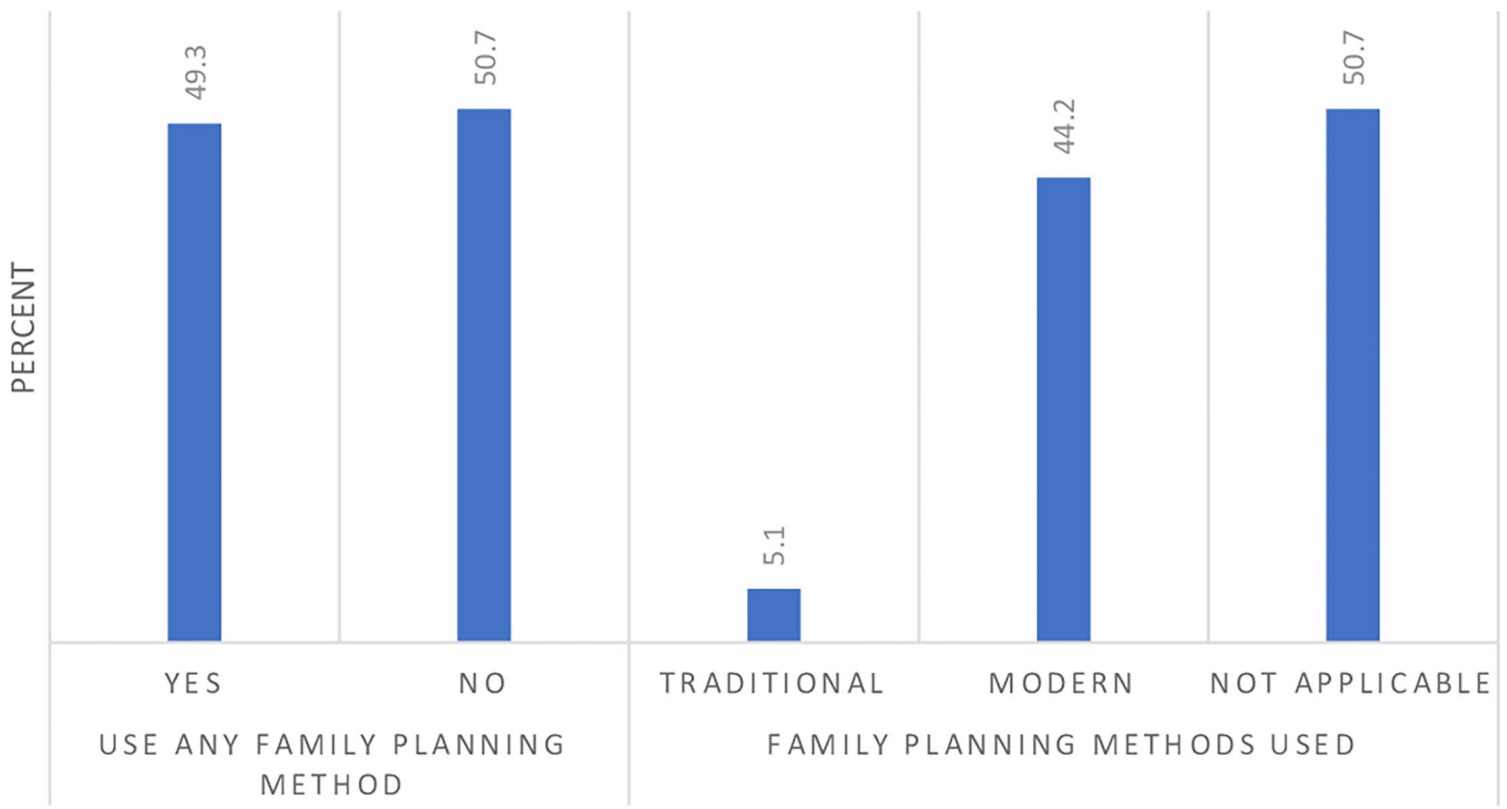
Use of family planning methods by the Ugandan youth during the COVID-19 lockdown.

Nearly half of the participants (49.3%) reported using family planning methods, the overwhelming majority of which used modern methods (Figure 1). Of the 320 participants using modern contraception, the majority (72.5%) used condoms, 10.3% reported using emergency pills, 6.9% relied on an IUD, 6.2% on an injectable contraceptive, and 4.1% used implants.

**Figure 2:**
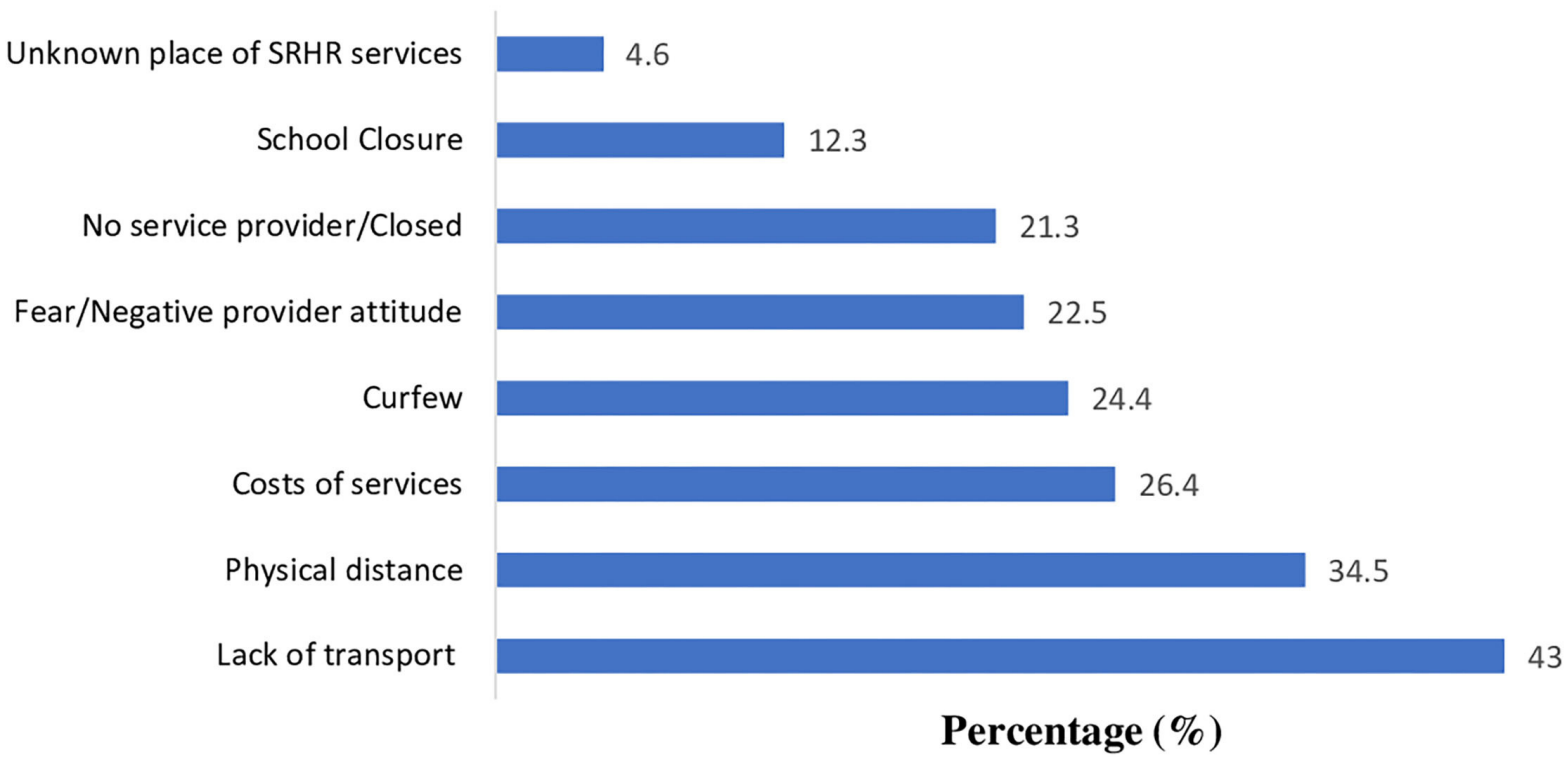
Limiting factors to access sexual and reproductive health services and information among Ugandan youth during the COVID-19 lockdown.

Lack of transport was the most commonly reported factor that impaired access to SRH services (43%), followed by distance from home (34.5%), cost of services (26.4%), and curfew (24.4%). Other factors were fear or/negative provider attitude (22.5%), no service provider at the facility (21.3%), school closure (12.3%), and lack of knowledge as to where SRH services could be obtained (Figure 2).

**Figure 3:**
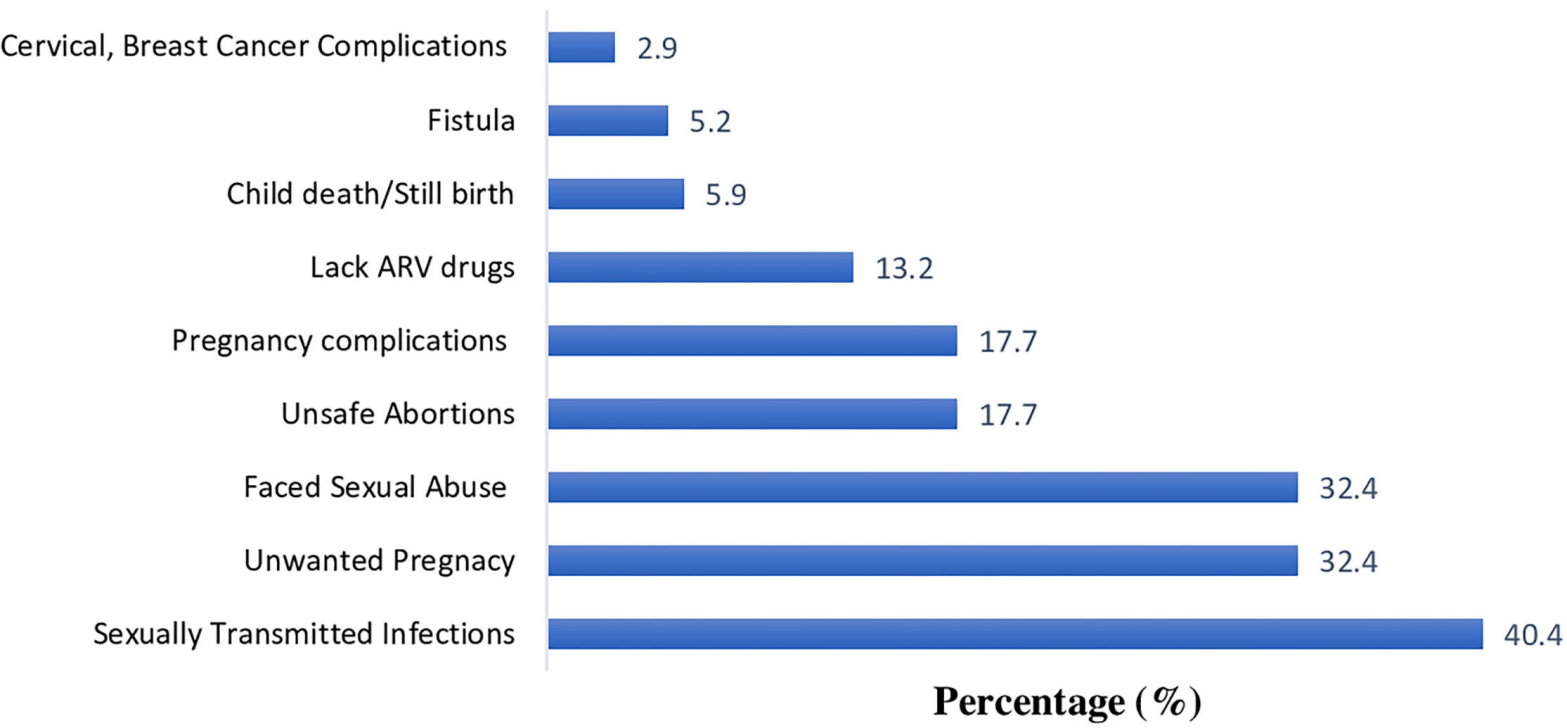
Problems associated with limited access to SRH information and services among Ugandan youth during the COVID-19 lockdown.

One hundred thirty-six of the participants (18.8%) reported having SRH challenges during the lockdown. Of these, STIs were the commonest problem (40.4%), followed by unwanted pregnancy (32.4%) and sexual abuse (32.4%). Other SRH problems included unsafe abortions (17.7%), pregnancy complications (17.7%), lack of anti-retroviral drugs (ARVs) (13.2%), death of a child (5.9%), and obstetrical fistula (5.2%) as shown in Figure 3.

### Relationship of Socio-Demographic Status

There was a statistically significant association between marital status and reports of difficulty in accessing SRH information and treatment (*p* < 0.001, Table 3). Cohabiting people [CPR: 1.3 (1.13–1.49)] were more likely to report limiting factors than single participants. Both bivariate and multivariate analyses indicated statistically significant association of educational status with reports of limiting factors to access to SRH information and services (*p* < 0.001). No statistically significant relationship was found for either geographic location or employment status, although there was a trend for.

**Table 3:** Bivariate and multivariate regression analyses using poisson regression of having a limiting factor to access SRH among Ugandan youth with their social demographics during the COVID-19 lockdown.

	**Bivariate**		**Multivariate**	
**Variable**	**CPR (95% CI)**	***P*-value**	**APR (95% CI)**	***P*-value**
**Sex**		0.965		**0.997**
Female	1		1	
Male	1 (0.89–1.12)		1 (0.89–1.12)	
**Age group in years**		0.740		**0.424**
18–24	1		1	
25–30	1 (0.91–1.14)		0.9 (0.83–1.08)	
**Marital status**		<0.001		**0.016**
Single	1		1	
Married	1.2 (1.04–1.41)		1.1 (0.97–1.36)	
Cohabiting	1.3 (1.13–1.49)		1.2 (1.06–1.41)	
**Education level**		<0.001		**0.001**
University	1		1	
Vocational or technical institution	1.4 (1.27–1.65)		0.9 (0.72–1.08)	
Secondary school	1.2 (1–1.45)		1.2 (0.94–1.48)	
**Location/region in Uganda**		0.088		**0.294**
Central Uganda	1		1	
Western Uganda	1 (0.91–1.2)		1 (0.9–1.2)	
Eastern Uganda	1.2 (1.03–1.4)		1.2 (0.99–1.34)	
Northern Uganda	1.1 (0.95–1.39)		1.1 (0.92–1.34)	
**Employment status**		0.048		**0.025**
Student	1		1	
Paid employment (employee on a salary)	1.1 (0.94–1.23)		1 (0.87–1.2)	
Self-employed (business/income generating activity)	1.2 (1–1.44)		1.1 (0.94–1.41)	
Unemployed: no structured activity	1 (0.79–1.22)		1 (0.79–1.21)	
Unemployed: volunteer/non-salaried	1.3 (1.09–1.53)		1.2 (1–1.42)	

*APR, Adjusted Prevalence ratio; CI, Confident Interval; CPR, Crude prevalence ratio. The bold values meaning with a p value of < 0.05 which are significant*.

### Association of Socio-Demographic Factors With the Reported SRH Health Challenges

Prevalence of SRH problems was significantly associated with marital status (*p* < 0.001, Table 4). People who co-habited [CPR: 2.7 (1.88–3.74) and APR: 2.3 (1.60–3.29)] were more likely to report problems, while single people were least likely. Education level also had a significant associated with, as university educated participants were less likely to report SRH challenges.

**Table 4:** Poisson regression of reported SRH problems on socio-demographic and economic factors with reported SRH problems among Ugandan youth during the COVID-19 lockdown.

	**Bivariate**		**Multivariate**	
**Variable**	**CPR (95% CI)**	***P*-value**	**APR (95% CI)**	***P*-value**
**Sex**		0.902		**0.994**
Female	1		1	
Male	1 (0.72–1.33)		1 (0.74–1.35)	
**Age group in years**		0.080		**0.661**
18–24	1		1	
25–30	1.3 (0.97–1.78)		1.1 (0.76–1.54)	
**Marital status**		<0.001		**<0.001**
Single	1		1	
Married	2 (1.38–3.02)		1.5 (0.99–2.32)	
Cohabiting	2.7 (1.88–3.74)		2.3 (1.60–3.29)	
**Education level**		<0.001		**0.001**
University	1		1	
Vocational or technical institution	2.3 (1.51–3.47)		0.5 (0.31–0.74)	
Secondary school	2.2 (1.47–3.4)		0.8 (0.45–1.33)	
**Location/region in Uganda**		0.306		**0.748**
Central Uganda	1		1	
Western Uganda	1.1 (0.74–1.56)		1.1 (0.75–1.53)	
Eastern Uganda	1.5 (0.97–2.2)		1.2 (0.82–1.79)	
Northern Uganda	1.1 (0.66–1.94)		0.9 (0.55–1.59)	
**Employment status**		0.034		**0.198**
Student	1		1	
Paid employment (employee on a salary)	1.5 (1.03–2.12)		1.2 (0.76–1.76)	
Self-employed (business/income generating activity)	1.7 (1.04–2.79)		1.2 (0.73–2.09)	
Unemployed: no structured activity	0.7 (0.32–1.41)		0.7 (0.33–1.44)	
Unemployed: volunteer or unpaid work	2 (1.27–3.2)		1.6 (1.03–2.64)	

*APR, Adjusted Prevalence ratio; CI, Confident Interval; CPR, Crude prevalence ratio. The bold values meaning with a p value of < 0.05 which are significant*.

## Discussion

In response to the COVID-19 Pandemic, Uganda introduced one of the most stringent lockdowns in Africa. The government banned public gatherings, shut down shopping centers, places of worship, schools and entertainment centers, restricted travel, and enacted a night-time curfew (15, 23). Although the travel ban was partially lifted for pregnant women and people with HIV/AIDS, the government did not address access to other essential SRH services such as contraceptives, menstrual sanitary supplies, and access to treatment for HIV (15, 17).

Many participants in this study reported poor access to SRH during the lockdown. Inadequate access was already a significant problem before the pandemic in Uganda as well as in many other developing countries (12–24). A 2015 study reported a significant unmet need for SRH services for young people in Wakiso district in Uganda (12). A qualitative study in the Kaborole district found widespread misinformation about contraception and STIs (25). Two major surveys in Uganda found that students, including those engaged in high-risk sexual behaviors, had limited access to SRH services and HIV/AIDS-related programmes (21–26). Despite efforts to improve access to SRH services, a survey of 70 resource poor countries found that <10% of adolescent women had access to health facilities and information about family planning (24). Inadequate access to SRH information and services has also been reported by studies in Kenya, Zambia, and Swaziland (27, 28).

Global health emergencies dictate shifts in priorities that present problems to the availability, accessibility, and affordability of SRH services (17). During previous epidemics and other outbreaks of infectious diseases in the past, poor access to SRH resources has been associated with increased maternal and childhood mortality (17). During West Africa's large multi-country Ebola Virus Disease (EVD) outbreak of 2014–2016, increased maternal mortality was attributed to closures of health facilities. Additionally, health care staff were reluctant to provide obstetrical care because infection control measures were inadequate, and they feared for their own safety (29). A study in Guinea found a decrease of 51% in Family Planning (FP) visits during an Ebola outbreak (30). Economic factors seemed to drive a spike in pregnancy that occurred during an Ebola outbreak in Liberia, as girls reportedly had sex in exchange for water, food, or other forms of financial protection (31).

In this study we found there was restricted access to family planning in Uganda during the COVID-19 lockdown. Nearly half of the participants reported the use of contraception. However, among those who used modern contraception, 59% had difficulty accessing their preferred method. Lack of transport was the commonest (68.7%) limiting factor to accessing SRH services and information during the lockdown. This was followed by physical distance from home to the health care facility (55.2%), cost of services (42.2%), and curfew (39.1%). Travel in motor vehicles, including private cars, taxis and buses, was banned in an effort to contain the spread of the COVID-19 in the community. Lack of motor transportation is compounded if the clinic is so far from home that walking is impractical. The lockdown also had an economic impact, as businesses closed, unemployment increased, and the cost of living increased. Thus, SRH services became less affordable. Inequalities in the disease burden and access to health care is prominent concern in Uganda and the economic impact of the shutdown could only exacerbate this problem (32).

STIs were the most commonly reported SRH problem. Lack of access to treatment is not only a problem for the infected individual. It is a public health problem due to potential spread of the disease. Participants also reported unwanted pregnancies, a significant public health problem that existed long before the pandemic. In 2008, more than half of the 2.2 million pregnancies in Uganda were unwanted (33). Teenage pregnancy has increased in Uganda during the COVID-19 lockdown (34). The closing of schools may have played a role in this increase, as girls are more vulnerable to abuse or rape outside the educational environment, which is more secure. It is also probable that a major additional factor was lack of access to SRH services, which are critical in the prevention of unwanted pregnancies, unsafe abortion, reducing maternal and child morbidity-mortality and empowering women (35).

This study had some limitations. In-depth interviews and focus group discussions to enrich the study findings were not possible, due to social distancing restrictions during the first wave of COVID-19 pandemic. The study was limited to young people who had access to internet and social media and an understanding of the English language. Furthermore, a respondent driven virtual snowball sampling method was used. Therefore, our findings may not be taken as a representation for the general Ugandan youth's population; our sample was skewed, as a very high percentage of participants had a university education. Finally, we do not have baseline comparison data from before the COVID-19 lockdown.

## Conclusion

In this study, young people in Ugandan reported poor access to sexual and reproductive health services during the COVID-19 lockdown. Lack of transportation and the price of services were the commonest factors reported as limiting access to SRH services. STIs and unwanted pregnancies were the most prevalent problems faced by Ugandan youth during the COVID-19 lockdown. There is a need for the Uganda government to collaborate with other stakeholders in developing guidelines for ensuring access to SRH information and services during any future outbreaks. Such guidance should include response plans for health facilities and the communities. New public health approaches are needed to strengthen supply chains and public information during crises that disrupt schools, health services, and community centers. Policymakers should define and promote sexual and reproductive health care as an essential service during any outbreak.

## Summary

The COVID-19 pandemic has disrupted access to healthcare, including sexual and reproductive health (SHR) services. An online cross-sectional study was conducted between 28th April and 11th May 2020 to explore factors affecting access to SRH services among Ugandan youth during the COVID-19 pandemic lockdown. In all, 724 Ugandan youth participated in the online survey. More than a quarter of respondents reported that during the lockdown, they had limited access to SRH services, such as testing and treatment for sexually transmitted infections (STIs), Human Immunodeficiency Virus (HIV) treatment, and contraception. Lack of transportation, distance to health facilities, and the high cost of services were the most commonly reported barriers to access. Marital and employment status as well as education level were statistically associated with the reported factors and problems to access SRH services. The most commonly reported SRH problems were STIs, followed by unwanted pregnancies, sexual abuse, unsafe abortions, pregnancy complications and lack of antiretroviral drugs. These findings indicate that effective measures need to be put in place to ensure access and availability of SRH services for Ugandan youth during outbreaks such as the COVID-19 lockdown.

## Data Availability Statement

The data used to obtain the findings is available from the corresponding author FS and the authors SBM and RS on a reasonable request.

## Ethics Statement

This study was approved by Kampala International University Research and Ethics Committee (KIU-REC-023/202018). Completion of a consent form was required as a key step to accessing the rest of the questionnaire and participation in the study. Participation in the study was voluntary and anonymous. Consent to participate was obtained through online acceptance.

## Author Contributions

SBM, FS, and RS were the principal investigators, conceived and designed the survey, supervised the online data collection, and critically reviewed the manuscript. YM analyzed data. FS, SAM, SA, and JN reviewed the manuscript development and revised the data tool. JR and FB revised the methodology. HW and LK participated in online data collection. FS, CK, and PK critically reviewed the manuscript. All authors read and approved the final manuscript.

## Conflict of Interest

The authors declare that the research was conducted in the absence of any commercial or financial relationships that could be construed as a potential conflict of interest.

## Publisher's Note

All claims expressed in this article are solely those of the authors and do not necessarily represent those of their affiliated organizations, or those of the publisher, the editors and the reviewers. Any product that may be evaluated in this article, or claim that may be made by its manufacturer, is not guaranteed or endorsed by the publisher.

## Abbreviations

APR: Adjusted Prevalence Ratio
CI: Confident Interval
COVID-19: Coronavirus Disease 2019
CSG: Coronavirus Study Group
DRC: Democratic Republique of the Congo
HIV: Human Immunodeficiency Virus
MOH: Ministry of Health
SARs: Severe Acute Respiratory Syndrome Coronavirus 2
SRH: Sexual and Reproductive Health
UBOS: Uganda Bureau of Statistics
WHO: World Health Organization.
