# Supplementary material for: Challenges in Access and Utilization of Sexual and Reproductive Health Services Among Youth During the COVID-19 Pandemic Lockdown in Uganda: An Online Cross-Sectional Survey
Source: Front Reprod Health. 2022 Feb 3;3:705609. doi: 10.3389/frph.2021.705609 (PMC9580707; doi:10.3389/frph.2021.705609)
Supplement: Supplementary file 1 [file Table_1.DOCX]

**Supplementary 1 Table: Questionnaire**

| 1. **Socio-demographic** | Questions | Answers |
| --- | --- | --- |
|  | Sex | Male  Female |
|  | Age | 18-24  25-30 |
|  | Marital Status | Single/Separated/Divorced  Married/Cohabiting |
|  | Education | College/University  Vocational or Technical Institution  Secondary School and below |
|  | Location | Central Uganda  Eastern Uganda  Western Uganda  Northern Uganda |
|  | Employment status | School  Paid employment (employee on a salary)  Self-employed (Business/Income Generating Activity)  Unemployed: No structured activity  Unemployed: Volunteer or unpaid work |
| 1. **Access to sexual and reproductive services of participants during the COVID-19 lockdown** | Is there any available information and/or education concerning sexuality given during COVID-19 pandemic (Lockdown)? | No  Yes |
|  | Are there testing and treatment services for SITs (Gonorrhoea, Chlamydia, syphilis, etc) during Lockdown? | No  Yes  Don’t know |
|  | How easily are you able to access your preferred modern contraceptive during the COVID-19 lockdown? | Not Easily  Easily  Not Applicable |
|  | Are HIV testing and Counselling services available during the COVID-19 lockdown? | No  Yes  Don’t know |
|  | Are you currently on HIV (ARVs) medication? | No  Yes |
|  | If yes, how easily are you able to access Antiretroviral therapy (medication) during the COVID-19 lockdown? | Not Easily  Easily  Not Applicable |
|  | If female, are you able to access menstrual health products such as sanitary pads? | Not Easily  Easily  Not Applicable |
|  | If you are pregnant or you have delivered, is pregnancy care available during the COVID-19 lockdown? | Yes  No |
|  | If you had abortion, are post abortion care services offered during the COVID-19 lockdown? | Yes  No |
|  | Do you use any family planning method? | Yes  No |
|  | If yes, which one are you using? | Modern  Traditional  Not applicable |
|  | If modern, which family planning method(s) are you using during the COVID-19 Lockdown? | Condom  Emergency pills  Injection  Implants  IUD  Not applicable |
| 1. **Limiting factors to access Sexual and reproductive health services and information during the COVID-19 lockdown** | Do you have difficult to access Sexual and reproductive health services and information during the COVID-19 lockdown? | Yes  No |
|  | What are factors which are limiting you to access Sexual and reproductive health services and information during the lockdown? (Select all that apply) | No service provider/Closed  Fear/Negative provider attitude  School Closure  Curfew  Fear/Negative provider attitude  Physical distance  Lack of transport  Costs of services  Unknown place of SRHR services |
| 1. **Problems related to SRHR during COVID-19 lockdown** | Have you heard any problem relating to sexual and reproductive health rights during the COVID-19 lockdown? | Yes  No |
|  | If yes, which problems have you faced during the COVID-19 lockdown? (Select all that apply) | Sexual Transmitted Infections  Faced Sexual Abuse  Unwanted Pregnancy  Unsafe Abortions  Pregnancies complications  Child death/Still Birth  Lack ARV drugs  Fistula  Cervical, Breast Cancer Complications |
